# Diabetic ketoacidosis and sinus arrest conditions in a patient with an inserted cardiac pacemaker

**DOI:** 10.1007/s00592-020-01615-4

**Published:** 2021-01-03

**Authors:** Takatoshi Anno, Kotone Tsujimoto, Ryo Shigemoto, Sunao Kojima, Hideaki Kaneto

**Affiliations:** 1 Department of General Internal Medicine 1, Kawasaki Medical School, 2-6-1 Nakasange, Kita-ku, Okayama, 700-8505 Japan; 2 Department of General Internal Medicine 3, Kawasaki Medical School, Okayama, 700-8505 Japan; 3 Department of Diabetes, Endocrinology and Metabolism, Kawasaki Medical School, Kurashiki, 701-0192 Japan

**Keywords:** Sinus arrest, Cardiac pacemaker, Diabetic ketoacidosis

## Introduction

It is well known in clinical practice that metabolic acidosis sometimes leads to life-threatening conditions. Acidosis itself is induced by various conditions and diseases, and is classified as either respiratory or metabolic acidosis.

Diabetic ketoacidosis (DKA), a form of metabolic acidosis, is one of the most serious acute metabolic complications of diabetes mellitus (DM), and is often observed in subjects with type 1 DM (T1DM). DKA is characterized by hyperglycemia, metabolic acidosis, and increased total ketone concentrations [1]. Here, we report a case with pre-existing T1DM and an inserted cardiac pacemaker. The patient with partial loss of consciousness was diagnosed with DKA, and she experienced sick sinus syndrome leading to sinus arrest in the presence of regular pulsation maintained by the cardiac pacemaker.

## Case Report

**Fig. 1:**
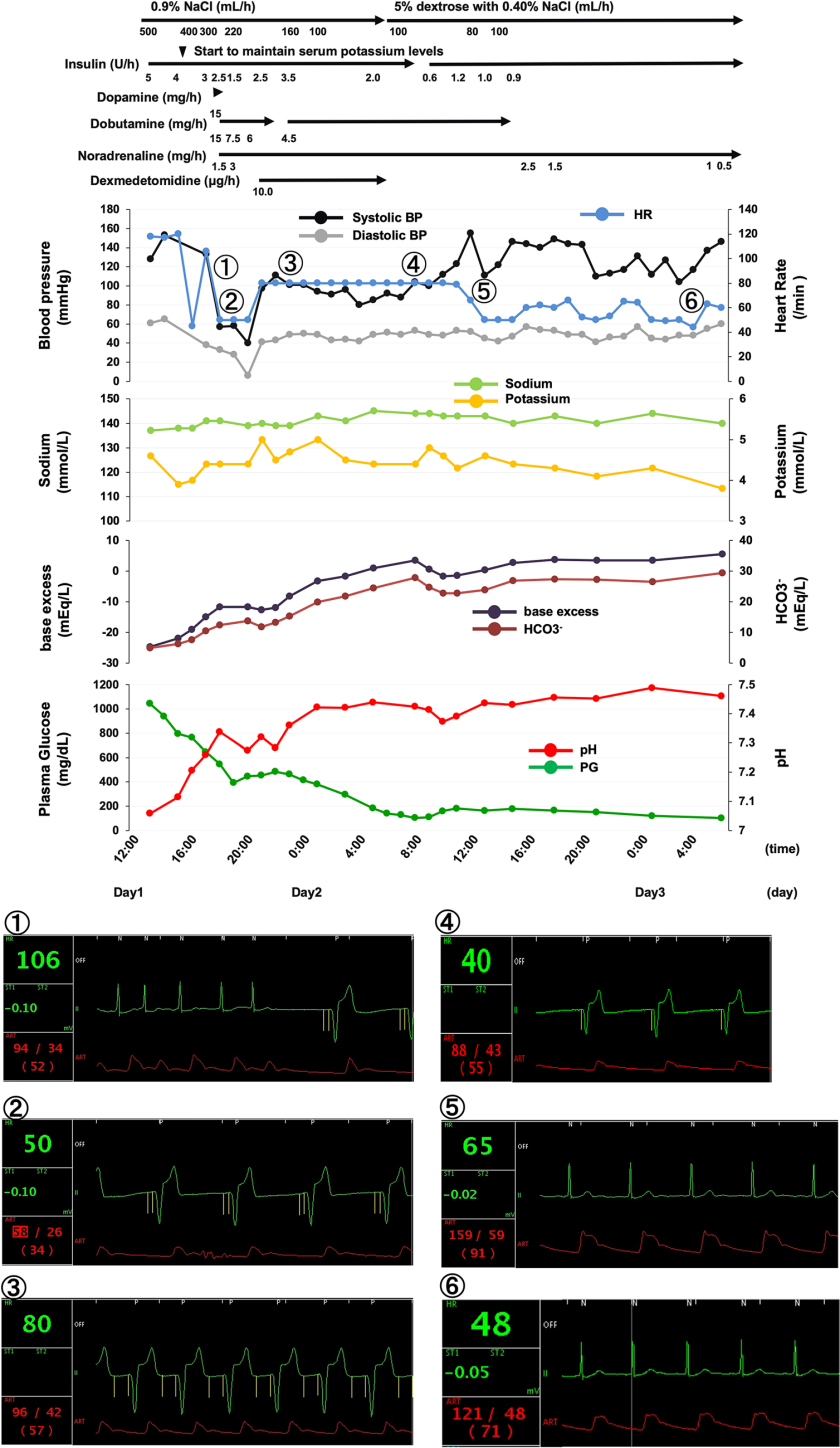
Clinical time course and biological monitor readings in the subject during DKA therapy period. To maintain serum potassium levels, 0.9% NaCl together with drip infusion and continuous insulin infusion were administered. Blood pressure suddenly and markedly decreased. Heart rate was 50 beats/min without fluctuation (①②). To maintain blood presser, the pacemaker was configured to 80 beats/min (③). The pacemaker was configured to 40 beats/min to confirm a spontaneous heartbeat (④). About 24 hours after a marked decrease of blood pressure, a spontaneous heartbeat was transiently observed by biological monitor (⑤). And about  hours later, a spontaneous heartbeat was steadily observed (⑥).

A 73-year-old woman with a 33-year history of T1DM visited the emergency room with symptoms of deteriorated consciousness. At 62 years of age a cardiac pacemaker was implanted for the treatment of sick sinus syndrome and paroxysmal atrial fibrillation. Following a diagnosis of dementia, glycemic control using insulin was poor and she was hospitalized three times due to DKA over the course of a single year. Upon admission, her vital signs were: temperature, 38.0 °C; blood pressure, 125/70 mmHg; heart rate, 138 bpm; oxygen saturation, 98%. Inflammation markers were markedly elevated (Table 1): white blood cell, 18900 /μL (neutrophil, 90.0%); C-reactive protein, 2.18 mg/dL; procalcitonin, 6.87 ng/mL. Liver function was close to normal, but renal function was elevated: creatinine, 2.02 mg/dL; blood urea nitrogen, 43 mg/dL. Above all, diabetes-associated data were markedly elevated; plasma glucose, 1044 mg/dL; hemoglobin A1c, 12.8%; glycoalbumin 55.6%. Ketone body concentrations were also markedly elevated: total ketone body, 26020.0 μmol/L; acetoacetate, 7000.0 μmol/L; *β*-hydroxybuterate, 19020.0 μmol/L. Blood gas analysis showed severe acidosis: pH 7.058; base excess (BE), − 24.7 mEq/L; HCO_3_^−^, 4.9 mEq/L (Fig. 1).

**Table 1:** Laboratory data observed in the emergence room

Variable	Result	Reference range	Variable	Result	Reference range
Peripheral blood			Dyslipidemia marker		
White blood cells (/μL)	18900	3300—8600	Total cholesterol (mg/dL)	139	142—248
Red blood cells ( × 10^4^/μL)	370	435—555	LDL cholesterol (mg/dL)	66	65—139
Hemoglobin (g/dL)	12.1	13.7—16.8	HDL cholesterol (mg/dL)	66	40—90
Platelets ( × 10^4^/μL)	22.4	15.8—34.8	Triglyceride (mg/dL)	239	40—149
Blood biochemistry			Diabetes marker		
Total protein (g/dL)	7.1	6.6—8.1	Plasma glucose (mg/dL)	1044	–
Albumin (g/dL)	3.9	4.1—5.1	Hemoglobin A1c (%)	12.8	4.9—6.0
Globulin (g/dL)	3.2	2.2—3.4	Glycoalbumin (%)	55.6	12.4—16.3
Total bilirubin (mg/dL)	0.4	0.4—1.5	Total ketone body (μmol/L)	26020.0	0.0—130.0
AST (U/L)	31	13—30	Acetoacetate (μmol/L)	7000.0	0.0—55.0
ALT (U/L)	17	10—42	*β*-Hydroxybuterate (μmol/L)	19020.0	0.0—85.0
LDH (U/L)	376	124—222	Blood Gas Analysis		
ALP (U/L)	367	106—322	pH	7.058	7.360—7.460
*γ*-GTP (U/L)	33	13—64	PCO2 (mmHg)	18.2	34.0—46.0
BUN (mg/dL)	43	8—20	PO2 (mmHg)	144.0	80.0—90.0
Creatinine (mg/dL)	2.02	0.65—1.07	HCO3- (mEq/L)	4.9	24.0—32.0
Cholinesterase (U/L)	525	240—486	BE (mEq/L)	-24.7	-2.5—2.5
Uric acid (mg/dL)	11.1	2.6—5.5	SO2 (%)	98.7	95.0—98.0
Creatine Kinase (U/L)	212	41—153	Urinary test		
Amylase (U/L)	1823	42—118	Urinary pH	5.0	5.0—7.5
P-Amylase (IU/L)	47	19—53	Urinary protein	-	–
CRP (mg/dL)	2.18	<0.14	Urinary sugar	3+	–
Procalcitonin (ng/mL)	6.87	0.00—0.05	Urinary ketone body	2+	–
Sodium (mmol/L)	134	138—145	Urinary bilirubin	–	–
Potassium (mmol/L)	4.5	3.6—4.8	Urinary blood	–	–
Chloride (mmol/L)	86	101—108	–	-	–
IP (mg/dL)	9.6	2.7—4.6	–	–	–
Magnesium (mg/dL)	3.1	1.9—2.6	–	–	–

*AST* aspartate aminotransferase, *ALT* alanine aminotransferase, *LDH* lactate dehydrogenase, *ALP* alkaline phosphatase, *γ*-GTP *γ*-glutamyltranspeptidase, *BUN* blood urea nitrogen, *P- Amylase* Pancreatic-Amylase, *CRP* C-reactive protein, *IP* Inorganic Phosphorus, *LDL* Low-density lipoprotein, *HDL* High-density lipoprotein, *BE* Base Excess

After admission, we administered 0.9% NaCl and gradually tapered total volume. We also maintained serum potassium levels with drip infusion and continuous, gradually tapered insulin infusion. Potassium levels were 4.5 mmol/L, within the mild-to-moderately elevated range of serum potassium seen under DKA conditions, and infusion of potassium was started after one point of observation to maintain serum potassium levels (Fig. 1). After approximately 5 hours, the patient’s blood pressure suddenly and markedly decreased to 58/26 mmHg. At that time, plasma glucose was decreased to 546 mg/dL and the blood gas analysis data improved (pH, 7.338). Mineral balance including potassium levels were within a normal range after therapy. Immediately prior to the decrease in blood pressure her heart rate was just 50 bpm without fluctuation, as determined by her cardiac pacemaker in DDD mode (see Fig. 1, ① and ②). Examination with echocardiography and abdominal enhanced computed tomography failed to detect any abnormalities such as ischemic heart disease, pulmonary thromboembolism and aortic dissection. We started vasopressors to rescue her from shock and used sodium bicarbonate, which is not used under DKA conditions except for severe DKA [1], because of the likelihood that the reduction of blood pressure and combined sinus arrest would be caused by DKA. To maintain her blood pressure within the minimum range, it was necessary to change the pacemaker configuration to 80 bpm (Fig. 1, ③). Upon vital sign stabilization, the pacemaker was temporarily configured to 40 bpm to confirm her spontaneous heartbeat (Fig. 1, ④). However, this test failed at that time. About 24 hours after the initial decrease in blood pressure, a spontaneous heartbeat was transiently detected (Fig. 1, ⑤). Finally, about 40 hours after the initial drop in blood pressure, the patient’s spontaneous heartbeat could be steadily detected (Fig. 1⑥). Over the 40 duration of this episode blood glucose levels, pH, base excess and HCO_3_^−^ gradually improved. Ketone body levels normalized 2 days later. With treatment and improvement of DKA conditions, the pacemaker-determined waveform was subsequently never observed.

## Discussion

DKA is the most serious acute metabolic complication of DM, sometimes leading to life-threatening situations. At 62-years-of-age, our patient was implanted with a cardiac pacemaker for the treatment of sick sinus syndrome. At the time of admission with partial consciousness followed by a sudden drop in blood pressure, we diagnosed and treated the patient for DKA-induced near-sinus arrest. We considered that the inserted cardiac pacemaker maintained a regular pulsation. In addition, the patient had no abnormalities such as ischemic heart disease, pulmonary thromboembolism and aortic dissection. This case report indicates that clinicians should know the possibility of DKA-induced critical conditions.

Under DKA conditions, sick sinus syndrome is aggravated by electrolyte abnormalities such as elevation of serum potassium. After intravenous insulin infusion to treat DKA, potassium replacement is required to prevent insulin therapy-induced hypokalemia. Therefore, both hyperkalemia and hypokalemia are closely involved in the development of arrhythmia and asystole under acidosis with DKA [2, 3]. It is also possible that hypophosphatemia is involved in the deterioration of sick sinus syndrome. Our patient fell into hypovolemic shock in association with DKA and sinus arrest, and after the treatment and recovery from DKA, hypovolemic shock did not recur. Although electrolyte imbalance in serum is not necessarily observed, intracellular electrolyte imbalance could be involved in the process. Therefore, it is likely that the intracellular electrolyte imbalance and metabolic acidosis led to the electrocardiogram abnormality which finally caused the reduction in heart rate and profound hypotension in this subject.

Moreover, if a patient with a pacemaker was complicated with hyperkalemia, hyperkalemia can cause loss of capture by a cardiac rhythm device without DKA [4]. Our patient’s initial potassium level was 4.4 mEq/L under sick sinus syndrome with an inserted cardiac pacemaker, although failure to capture is usually seen when potassium level reaches around 7 mEq/L. The American Diabetes Association Statements show that it is appropriate to start 20–30 mEq potassium in each liter of intravenous fluid in order to keep serum potassium between 4–5 mEq/L when starting potassium level is 3.3–5.2 mEq/L [1]. In emergency room, her potassium level was 4.6 mmol/L, and we started 0.9% NaCl and transferred her to a high care unit. About 2 hours later her potassium level was 3.9 mmol/L, and we started infusion of potassium with 20 mEq potassium (potassium L-asparta or potassium chloride). We assume that intracellular electrolyte imbalance was, at least in part, associated with sinus arrest in this subject. On the other hand, since it is known that dexmedetomidine causes atrioventricular block and sinus arrest, it is possible that dexmedetomidine led to deterioration of heart function [5].

Taken together, it is likely that DKA was closely associated with sick sinus syndrome and that the intracellular electrolyte imbalance and metabolic acidosis led to the electrocardiogram abnormality which finally caused the reduction in heart rate and profound hypotension in this subject. We should bear in mind the possibility that DKA by electrolyte imbalance causes deterioration of sick sinus syndrome leading to prolonged sinus arrest.
